# Physical fitness components as mediators between physical activity and executive function in adolescents: a cross-sectional study

**DOI:** 10.3389/fpubh.2026.1824502

**Published:** 2026-07-15

**Authors:** Wenqing Duan, Huipan Wu, Jian Wu, Jinxian Wang

**Affiliations:** 1 School of Physical Education, North University of China, Taiyuan, China; 2 Research Center for Health Promotion of Children and Adolescents, Taiyuan Institute of Technology, Taiyuan, China; 3 School of Physical Education, Shanxi University, Taiyuan, China

**Keywords:** adolescents, China, executive function, physical activity, physical fitness

## Abstract

**Purpose:**

This study examined whether physical fitness (PF) mediates the association between physical activity (PA) and executive function (EF) in Chinese adolescents.

**Methods:**

A cross-sectional study was conducted from September to December 2023, involving 5,713 students aged 13–18 years recruited through stratified cluster random sampling. PA was assessed using questionnaires, PF through standardized field tests, and EF using computerized cognitive tasks. Partial correlation analyses and mediation models were performed.

**Results:**

No significant sex differences were observed in inhibitory control or the 1-back task. Moderate-to-vigorous PA (MVPA) correlated positively with most PF indicators (*p* < 0.05). PF indicators were generally negatively correlated with EF measures (*p* < 0.05). The total effect of MVPA on 1-back performance was not significant (*p* = 0.109). However, in separate simple mediation models, MVPA showed significant specific indirect associations with working memory via core strength (indirect effect = −0.030), cardiorespiratory fitness (indirect effect = −0.021), and agility and coordination (indirect effect = −0.023) (95% CIs did not include 0; *p* < 0.05). In stratified analyses, indirect effects were significant in females, adolescents with normal weight, and those meeting screen-time guidelines, but not in males, senior high school students, adolescents with obesity, or those exceeding screen-time limits.

**Conclusion:**

PF (specifically core strength, cardiorespiratory fitness, and agility and coordination) exerts an inconsistent (suppression) mediation effect in the relationship between MVPA and working memory in Chinese adolescents.

## Introduction

Physical activity (PA) is well known to promote neurocognitive development and contribute to the enhancement of executive function (EF) in children and adolescents (1, 2), although the mechanisms through which PA influences cognitive outcomes remain incompletely understood (3). It represents a promising behavioral approach for enhancing cognitive function. However, recent scientific models have highlighted the limitations of focusing solely on behavioral volume and have recommended that researchers investigate physical fitness (PF), the physiological adaptation to moderate-to-vigorous physical activity (MVPA), as a more enduring cognitive driver (4). EF, defined as a set of higher-order cognitive processes used to engage, guide, and coordinate lower-order cognitive processes (5, 6), has been shown not only to improve in association with PA but also to support performance on tasks that depend on efficient executive control (7). A growing body of work suggests that PF, PA, and physical activity interventions are beneficial for children’s cognitive function (8–10). Among the various cognitive domains, EF appears to exhibit the strongest associations with both PF and PA (11). Inhibitory control, working memory, and cognitive flexibility—the three core subdomains of EF—are particularly important for adolescents’ daily functioning, physical and mental development, and social adaptation (12, 13).

While intervention studies and meta-analyses have consistently highlighted the benefits of PA on overall EF (14–17), findings from previous research have primarily focused on the behavioral volume itself. Currently, PF is increasingly proposed as a working model representing physiological adaptation (8), and the mechanisms through which PA and PF influence EF may differ (11). PA reflects a behavioral process that may exert acute and transient effects on cognition (18), whereas PF represents a relatively stable physiological state that may provide a more enduring foundation for cognitive development (11). Nevertheless, research distinguishing the independent contributions of PA and PF to EF remains limited. Moreover, although health-related fitness has frequently been examined as a single global indicator, evidence regarding the specific associations between skill-related dimensions of PF and individual EF subdomains remains scarce, particularly for agility and coordination, which are characterized by substantial cognitive engagement through participation in open-skill activities (19–24).

An important approach to examining the role of a variable in the association between other constructs is mediation analysis, which tests whether the effect of a predictor on an outcome is transmitted through an intermediate variable (mediator) (25). This approach has been applied in adolescent populations to investigate the mediating role of PF in the relationship between PA and EF (26), as well as academic achievement (27). These studies reported that, when specific components of PF were included as mediators, cardiorespiratory fitness partially mediated the association between MVPA and cognitive flexibility, whereas muscular strength mediated the associations between MVPA and both inhibitory control and cognitive flexibility. However, these findings are constrained by incomplete assessment of PF (largely omitting skill-related components) and a relatively narrow set of measurement dimensions, which limits a more comprehensive understanding of how a broader, multi-component PF framework may shape the relationship between PA and EF. Based on this framework, we hypothesized that different dimensions of PF would exert distinct and parallel mediating effects in the association between MVPA and EF. Specifically, we expected metabolic adaptations (e.g., cardiorespiratory fitness), muscular strength (e.g., core strength), and skill-related adaptations (e.g., agility and coordination) to emerge as independent mediators. Notably, although our initial assessment included three core EF subdomains (inhibitory control, working memory, and cognitive flexibility), preliminary analyses indicated that working memory was the only subdomain showing robust and consistent associations with the fitness indicators. Therefore, the results of this study specifically focus on working memory to ensure a targeted and rigorous interpretation of the significant pathways observed.

To address these knowledge gaps, this study adopted a multi-component PF perspective to disentangle the mechanisms underlying the association between PA and EF in Chinese adolescents, and to examine the mediating role of PF in this association, thereby contributing to the field of public health.

## Methods

### Study design

**Figure 1:**
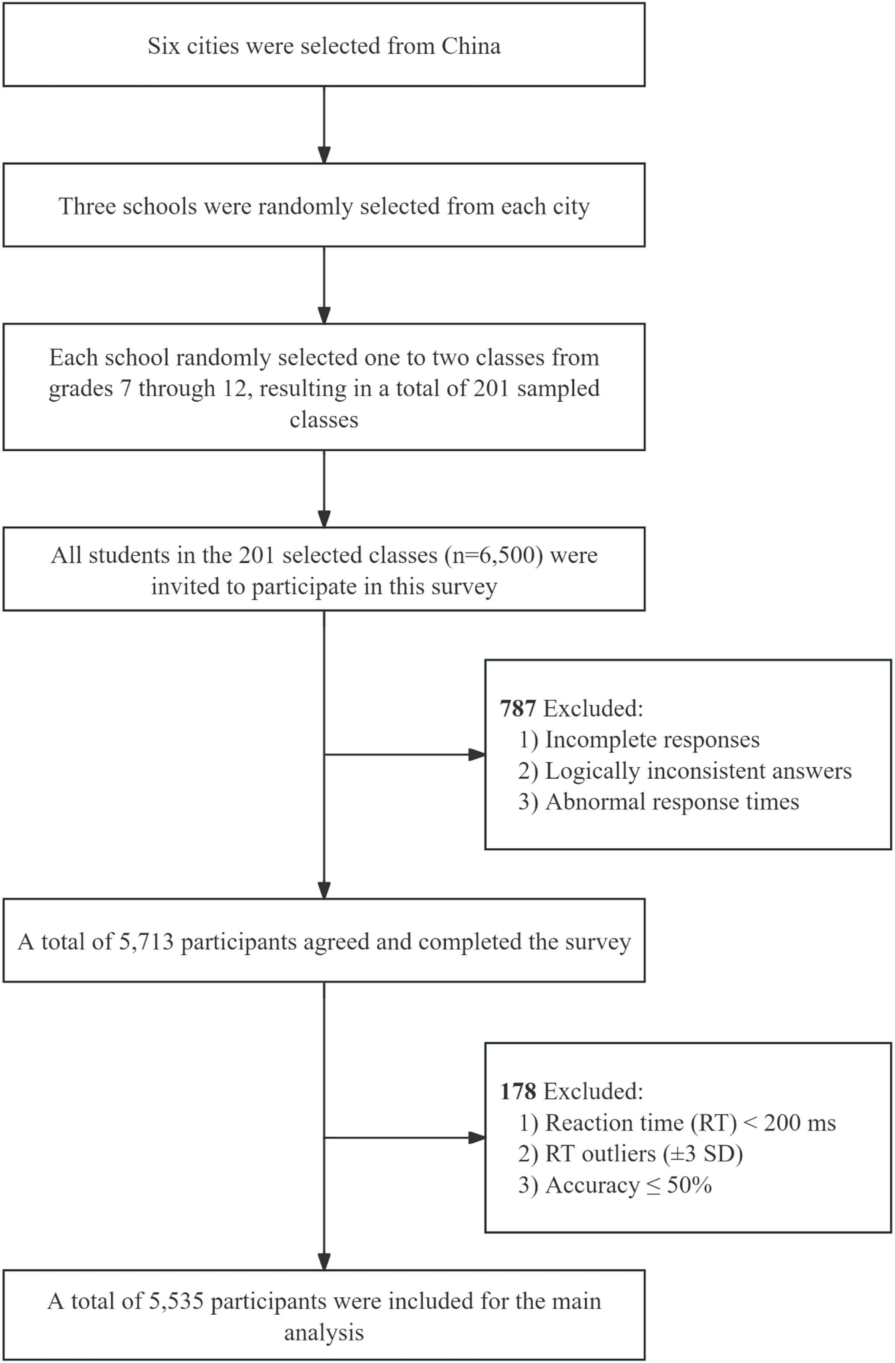
Flow diagram of participant inclusion process.

**Figure 2:**
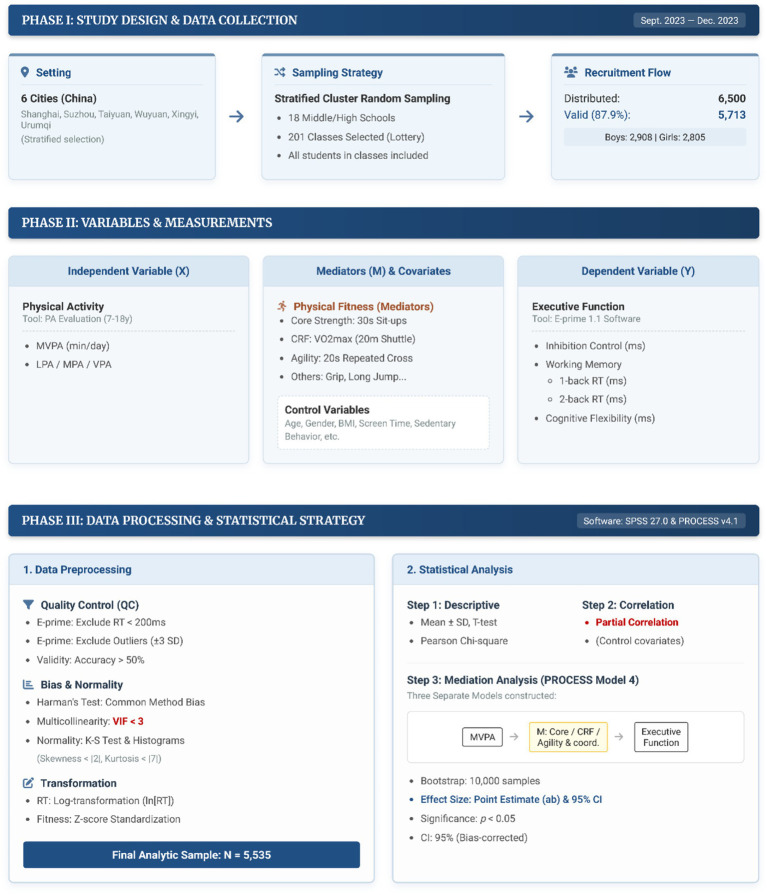
Research flow chart.

We conducted a school-based cross-sectional survey between September and December 2023. Using a stratified cluster random sampling design, questionnaires were administered in six cities (Shanghai, Suzhou, Taiyuan, Wuyuan, Xingyi, and Urumqi), with stratification by sex and age. First, three secondary schools were randomly selected in each city. Within each school, 1–2 classes from grades 7 to 12 were randomly selected, resulting in 201 classes. Each class was treated as a sampling unit, and all students who provided informed consent were invited to participate. Paper-based questionnaires were administered in classrooms. A total of 6,500 questionnaires were distributed, and 5,713 valid responses were collected, yielding a valid response rate of 87.9%. Invalid questionnaires were excluded based on the following criteria: (i) incomplete responses; (ii) logically inconsistent responses; and (iii) abnormal response times. Prior to formal analysis, an additional 178 participants were excluded according to the following criteria: (i) reaction time < 200 ms; (ii) values exceeding ±3 SD; and (iii) accuracy ≤ 50%. The final analytic sample comprised 5,535 adolescents (2,778 males and 2,757 females; 3,278 junior high school students and 2,257 senior high school students), with a mean age of 15.11 ± 1.70 years. See Figure 1 for the screening process. This study was conducted in accordance with the Declaration of Helsinki and approved by the Human Research Ethics Committee of East China Normal University (Approval No. HR761-2022). Written informed consent was obtained from all participants and their parents or legal guardians. The study flow is shown in Figure 2.

### Physical activity

PA was assessed using the Physical Activity Level Evaluation for Children and Adolescents Aged 7–18 Years (28). This questionnaire assesses the type, frequency, duration, and perceived intensity of PA. Based on metabolic equivalents (METs), PA was categorized into three intensity levels: light physical activity (LPA; 1.5 to <3 METs), moderate physical activity (MPA; 3 to <6 METs), and vigorous physical activity (VPA; ≥6 METs). Total weekly PA time was calculated by multiplying frequency by the average duration of each session. In Chinese populations, this instrument has demonstrated acceptable concurrent validity relative to the original validated version (*r* = 0.689), as well as moderate test–retest reliability (*r* = 0.606) (29).

### Anthropometric variables

Anthropometric measurements were conducted in accordance with the National Student Physical Fitness Standard (2014) (30). Participants wore light clothing and no shoes during testing. Height and body mass were measured using standardized equipment, while waist circumference was assessed with a non-elastic measuring tape positioned horizontally around the abdomen at the midpoint between the lower rib margin and the iliac crest. Height, body mass, and waist circumference were recorded to one decimal place. Body mass index (BMI) was calculated as body mass (kg) divided by height squared (m^2^).

### Physical fitness

**Figure 3:**
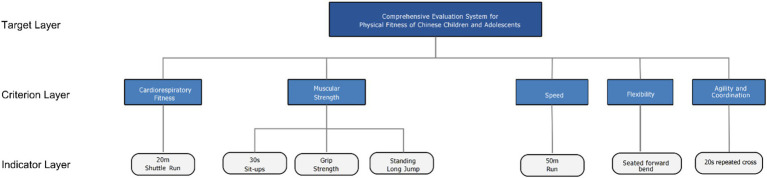
Comprehensive evaluation system of physical health of children and adolescents in China.

PF was assessed using the New Evaluation Standards for the Physical Fitness of Chinese Children and Adolescents (31). This assessment system was developed based on extensive literature review, empirical validation, and expert consultation and is designed to comprehensively evaluate the major dimensions of youth PF, including cardiorespiratory fitness, muscular strength, flexibility, speed, and agility-coordination. Several measures included in this battery, such as the 20-m shuttle run, handgrip strength, standing long jump, and sit-up tests, are also widely used in established international youth fitness assessment systems, including FITNESSGRAM and EUROFIT, supporting their scientific validity and cross-population comparability. Specifically, the 20-m shuttle run was used to assess cardiorespiratory fitness; handgrip strength, 30-s sit-ups, and standing long jump were used to evaluate upper-body strength, core muscular endurance, and lower-body explosive strength, respectively; the 50-m sprint served as an indicator of speed; the seated forward bend assessed flexibility; and repeated crossovers were used to evaluate agility and coordination. It should be noted that the repeated crossover test reflects a combined agility–coordination capacity rather than assessing these two constructs separately. As shown in Figure 3.

#### 20-meter shuttle run

Participants ran back and forth between two lines 20 meters apart in time with audio signals. The test was terminated when a participant was unable to maintain the required pace or failed to reach the line twice consecutively before the end of the audio signal. The final score was the total number of completed laps.

#### Grip strength

Handgrip strength was measured using an electronic dynamometer (AC3249). Participants performed two maximal handgrip trials, and the highest value was recorded in kilograms to two decimal places.

#### 30-s sit-ups

The total number of sit-ups completed within 30 s, with elbows required to touch or move beyond the knees, was recorded.

#### Standing long jump

The best distance from three bilateral standing long jump trials was recorded in centimeters to two decimal places.

#### 50-meter run

The time required to complete a 50-meter sprint from the starting signal was recorded in seconds to two decimal places.

#### Seated forward bend

The maximal distance participants could push a test board forward with both arms while seated with extended legs was recorded in centimeters to two decimal places.

#### 20 s repeated cross

The number of side-to-side crossings completed within 20 s was recorded, with each full cycle (i.e., returning to the center line from both sides) counted as four movements.

The adopted fitness battery was derived from the New Evaluation Standards for the Physical Fitness of Chinese Children and Adolescents (31), which were developed using a nationwide sample of more than 100,000 children and adolescents from 26 provinces in China. All assessments were conducted by trained personnel following standardized testing procedures, with pilot testing, supervision, and quality-control measures implemented throughout data collection to ensure measurement consistency and data quality.

### Executive function tests

The EF task paradigm comprised the inhibitory control task designed by Flanker, a working memory task, and a cognitive flexibility task (32). Both response accuracy and reaction time (RT) for correct responses were recorded as the primary performance indicators, with higher accuracy and shorter RTs reflecting superior EF. The tasks were programmed and administered on a computer using E-prime 1.1 software (Psychology Software Tools Inc., Pittsburgh, PA, United States).

#### Inhibition control

According to the Flanker experiment protocol requirements (33), the inhibitory function test primarily consists of two parts: the congruent response test and the incongruent response test. Each trial began with a central fixation cross presented for 500 ms, followed by a horizontal string of five uppercase letters presented for 1,000 ms. In congruent conditions, the letters were identical (e.g., LLLLL or FFFFF). In incongruent conditions, the central target letter differed from the flanking letters (e.g., LLFLL or FFLFF). These two conditions appeared randomly. Participants were instructed to respond to the central target letter by pressing the “F” key if it was “F,” and the “L” key if it was “L,” using their left or right index fingers. The task consisted of a practice phase containing 12 trials, followed by two formal blocks of 48 trials each. Inhibitory control capacity was evaluated using the RT difference between the incongruent and congruent conditions.

#### Working memory

Working memory was evaluated using the 1-back and 2-back tasks. In the 1-back task, a sequence of five uppercase letters (e.g., A, S, P, G, T) was presented centrally. Participants were required to rapidly judge whether the current letter matched the immediately preceding letter, pressing the “F” key for a match and the “J” key for a mismatch. Each letter remained visible for 2,000 ms, with an inter-stimulus interval (ISI) of 3,000 ms. The task consisted of two blocks, each containing 25 trials. The 2-back task followed identical timing and procedural parameters, except participants were required to judge whether the current letter matched the one presented two trials prior.

#### Cognitive flexibility

The cognitive flexibility assessment was divided into three parts involving numbers from 1 to 4 and 6 to 9. In the first part (Block A, 16 trials), participants evaluated the magnitude of randomly appearing black numbers, pressing “D” for numbers < 5 and “F” for numbers > 5. In the second part (Block B, 16 trials), participants evaluated the parity of green numbers, pressing “J” for odd numbers and “K” for even numbers. Blocks A and B constituted the homogeneous conditions. In the third part (Block C, 32 trials), the magnitude and parity tasks were randomly interleaved (heterogeneous condition). When a black number appeared, participants applied the magnitude rule; when a green number appeared, they applied the parity rule. The formal testing followed an A-B-C-C-B-A block sequence. Unscored practice rounds consisted of 8 trials before the first and second parts, and 16 trials before the third part. Cognitive flexibility was evaluated using the RT difference between the heterogeneous and homogeneous conditions.

#### Statistical analysis

The normality of the variable distributions was tested using both statistical (Kolmogorov–Smirnov test combined with skewness and kurtosis) and graphical methods (normal probability plots). To correct for positive skewness and meet the normality assumptions for parametric testing, variables including PA volume (e.g., MVPA) and EF reaction times (RT) underwent natural logarithmic (Ln) transformation prior to inferential analyses.

Adolescents’ characteristics are presented as the mean ± SD and the frequency (%) for continuous and categorical variables, respectively. The Student *t*-test and the Pearson’s χ^2^ test were used to test sex differences in relation to adolescents’ PA, PF, and EF. Pearson partial correlation coefficients (*r*) were used as a preliminary analysis to examine the associations among PA, PF, and EF. Multicollinearity was tested before completing the mediation analysis through tolerance values and variance inflation factors (VIF), ensuring acceptable limits.

To test the mediating role of PF in the association between MVPA and EF, we conducted mediation analyses using the PROCESS macro (Model 4) for SPSS developed by Hayes. Specifically, separate simple mediation models were constructed for each of the assessed PF components to independently evaluate their specific indirect effects. The significance of the indirect effects was tested using a bias-corrected bootstrapping approach with 10,000 resamples; an effect was considered significant if the 95% confidence interval (CI) did not include zero. Pathways that yielded statistically significant mediation effects (e.g., core strength, cardiorespiratory fitness, and agility and coordination) were subsequently detailed in the results. Crucially, all partial correlations and mediation models consistently adjusted for the same set of covariates: age, sex, BMI, screen time, and sleep quality. Statistical significance was set at *p* < 0.05.

## Results

Table 1 shows the characteristics of the study population. The partial correlation coefficients among PA, PF, and EF are presented in Table 2. It should be explicitly noted that this bivariate correlation matrix was treated strictly as an exploratory preliminary step, whereas the subsequent mediation analyses served as our confirmatory tests. There was no difference in inhibition control and 1-back reaction time between sexes (*p* > 0.05). Importantly, higher PF levels were significantly associated with shorter reaction times (i.e., better EF performance) across most EF indicators (*p* < 0.05), with the exception of standing long jump and grip strength; grip strength was not associated with any EF indicators. MVPA was associated positively with most PF indicators (*p* < 0.05), except for standing long jump, seated forward bend, and 50-meter run. We observed no severe multicollinearity; the tolerance values ranged from 0.476 to 0.971, and the variance inflation factors ranged from 1.030 to 2.102.

**Table 1:** Descriptive characteristics of the study population.

Variables	Overall (*n* = 5,535)	Boys (*n* = 2,778)	Girls (*n* = 2,757)	Effect size
Age (year)	15.1 ± 1.7	15.1 ± 1.7	15.1 ± 1.7	−0.01
Body mass (kg)	59.9 ± 15.1	63.5 ± 16.2	56.2 ± 13.0∗	0.48
Height (cm)	165.8 ± 8.9	169.1 ± 9.2	162.3 ± 7.0∗	0.81
BMI (kg/m2)	21.7 ± 4.6	22.1 ± 4.6	21.3 ± 4.4∗	0.16
Waist circumference (cm)	72.0 ± 11.1	75.3 ± 11.9	68.6 ± 9.0∗	0.63
Screen time (h/day)	1.2 ± 1.6	1.2 ± 1.6	1.1 ± 1.6∗	0.12
Sedentary time (h/day)	10.4 ± 4.1	10.3 ± 4.1	10.6 ± 4.0∗	−0.01
LPA (min/day)	28.0 ± 44.0	32.1 ± 47.4	23.9 ± 40.0∗	0.18
MPA (min/day)	45.2 ± 51.4	48.4 ± 58.4	42.0 ± 43.0∗	0.13
VPA (min/day)	36.5 ± 42.7	42.3 ± 48.2	30.6 ± 35.4∗	0.28
MVPA (min/day)	81.7 ± 77.0	90.7 ± 86.6	72.6 ± 64.8∗	0.24
MVPA met standard (yes, %)	2,464 (44.5%)	1,375 (49.5%)	1,089 (39.5%)∗	0.10
VO2max (ml/kg/min)	46.3 ± 8.2	50.3 ± 8.2	42.2 ± 5.9∗	1.12
Grip strength (kg)	28.3 ± 10.5	32.6 ± 10.8	23.9 ± 8.0∗	0.91
Standing long jump (cm)	183.0 ± 33.7	200.0 ± 31.6	164.8 ± 25.4∗	1.22
30-s sit-ups (n)	22.3 ± 9.1	23.4 ± 9.0	21.2 ± 9.1∗	0.19
Seated forward bend (cm)	11.2 ± 19.0	9.6 ± 20.6	12.7 ± 17.1∗	−0.13
50 m run (s)	7.0 ± 4.7	6.7 ± 4.3	7.3 ± 5.0∗	0.01
20 m shuttle run (laps)	40.7 ± 19.6	47.5 ± 21.4	33.8 ± 14.7∗	0.72
20 s repeated cross (n)	27.7 ± 12.8	29.4 ± 13.4	26.0 ± 12.0∗	0.26
Inhibition control (ms)	2.9 ± 34.5	3.7 ± 34.4	2.2 ± 34.6	0.04
Flanker accuracy (%)	87.3 ± 18.3	86.9 ± 18.6	87.6 ± 18.0	0.01
1-back (ms)	846.5 ± 333.3	841.0 ± 336.6	852.0 ± 329.8	−0.01
1-back accuracy (%)	75.8 ± 23.8	75.1 ± 23.9	76.5 ± 23.8*	−0.06
2-back (ms)	1049.3 ± 377.2	1031.0 ± 380.8	1067.7 ± 372.6∗	−0.09
2-back accuracy (%)	61.4 ± 20.1	60.4 ± 19.5	62.5 ± 20.6*	0.01
Cognitive flexibility	298.4 ± 174.2	278.4 ± 172.5	318.6 ± 173.7∗	−0.23
Cognitive flexibility accuracy (%)	85.2 ± 13.6	84.4 ± 14.2	86.0 ± 12.9*	−0.11

Values are presented as mean ± SD for continuous variables or frequency (%) for categorical variables. Independent two-tailed *t*-tests or chi-square tests were used to compare differences between genders. Standardized effect sizes for group differences are reported as Cohen’s d for continuous variables and Cramer’s V for categorical variables. ^⁎^*p* < 0.05, compared with boys.

**Table 2:** Partial correlation coefficients (r) among physical activity, physical fitness, and cognitive function.

Variables	LPA	MPA	VPA	MVPA	Grip strength	Standing long jump	30-s sit-ups	Seated forward bend	20 m shuttle run	20s repeated cross	50 m run	Inhibition control	1-back	2-back	Cognitive flexibility
LPA	–	–	–	–	–	–	–	–	–	–	–	–	–	–	–
MPA	0.03	–		–	–	–	–	–	–	–	–	–	–	–	–
VPA	0.15*	0.31*	–	–	–	–	–	–	–	–	–	–	–	–	–
MVPA	0.10*	0.85*	0.76*	–	–	–	–	–	–	–	–	–	–	–	–
Grip strength	0.03	0.04*	0.06*	0.06*	–	–	–	–	–	–	–	–	–	–	–
Standing long jump	0.01	−0.04	0.01	−0.02	0.14*	–	–	–	–	–	–	–	–	–	–
30-s sit-ups	0.02	0.04*	0.04*	0.05*	0.22*	0.20*	–	–	–	–	–	–	–	–	–
Seated forward bend	−0.02	−0.02	−0.01	−0.02	0.05*	0.08*	0.04*	–	–	–	–	–	–	–	–
20 m shuttle run	−0.01	0.02	0.04*	0.04*	0.12*	0.09*	0.19*	0.07*	–	–	–	–	–	–	–
20s repeated cross	0.06*	0.03*	0.03*	0.04*	0.10*	0.04*	0.36*	0.04*	0.13*	–	–	–	–	–	–
50 m run	−0.02	−0.04	0.01	−0.02	0.03*	0.48*	0.07*	0.35*	0.06*	0.01	–	–	–	–	–
Inhibition control	0.03*	−0.02	−0.01	0.02	−0.02	−0.01	0.01	0.01	−0.01	0.02	−0.01	–	–	–	–
1-back	0.00	0.02	0.02	0.02	0.02	0.13*	−0.13*	−0.05*	−0.13*	−0.14*	0.03*	−0.04*	–	–	–
2-back	0.02	−0.01	−0.01	−0.01	−0.02	0.09*	−0.16*	−0.08*	−0.18*	−0.07*	−0.10*	−0.02	0.53*	–	–
Cognitive flexibility	0.04*	−0.04*	−0.01	0.03*	−0.01	0.06*	−0.03	−0.05*	−0.09*	0.01	−0.10*	−0.03*	0.19*	0.34*	–

Model was adjusted for age, sex.

^⁎^*p* < 0.05, significant association.

**Figure 4:**
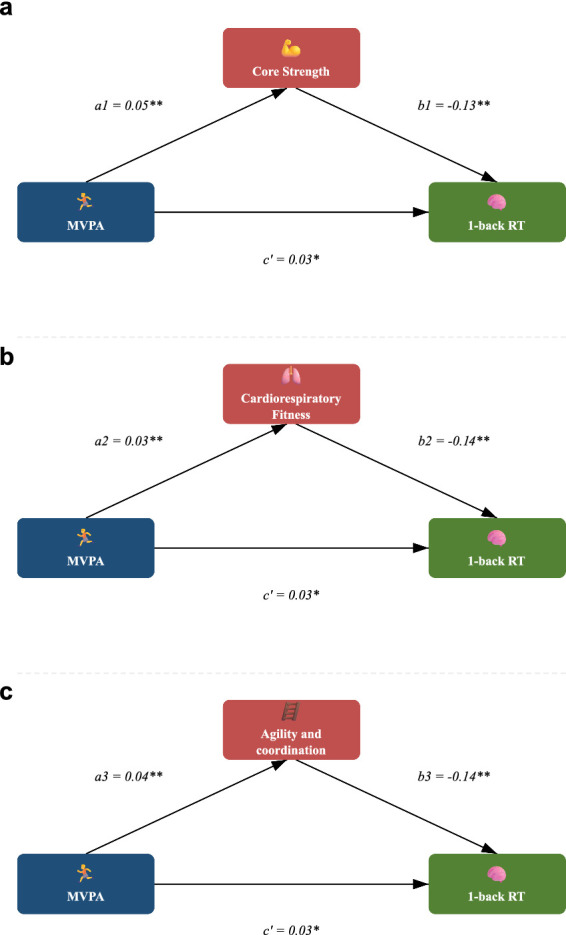
Mediation analysis. Contribution of PA on EF through physical fitness components, adjusting for potential confounders (age, sex, BMI, screen time, and sleep quality). **(A)** Core strength as a mediator. **(B)** Cardiorespiratory fitness as a mediator. **(C)** Agility and coordination as a mediator.

The results from the mediation analyses are shown in Figure 4. Overall, the total effect of MVPA on 1-back reaction time was not significant (*p* > 0.05). However, upon controlling for PF mediators, the direct effects (c’) of MVPA on 1-back reaction time emerged as positive and significant (*p* < 0.05), whereas the specific indirect effects through PF were significant and negative. Specifically, MVPA indirectly promoted faster 1-back reaction times via enhanced core strength (indirect effect = −0.030; SE = 0.011; 95% CI: −0.053, −0.011), cardiorespiratory fitness (indirect effect = −0.021; SE = 0.010; 95% CI: −0.042, −0.003), and agility and coordination (indirect effect = −0.023; SE = 0.009; 95% CI: −0.043, −0.006). Statistically, the opposing directions of the direct (positive) and indirect (negative) effects indicate a competitive mediation, or suppression effect. The overall simple mediation models, adjusting for covariates, accounted for significant proportions of the variance in working memory performance. Specifically, the independent models incorporating core strength, cardiorespiratory fitness, and agility and coordination yielded *R*^2^ values of 0.019, 0.019, and 0.021, respectively.

**Figure 5:**
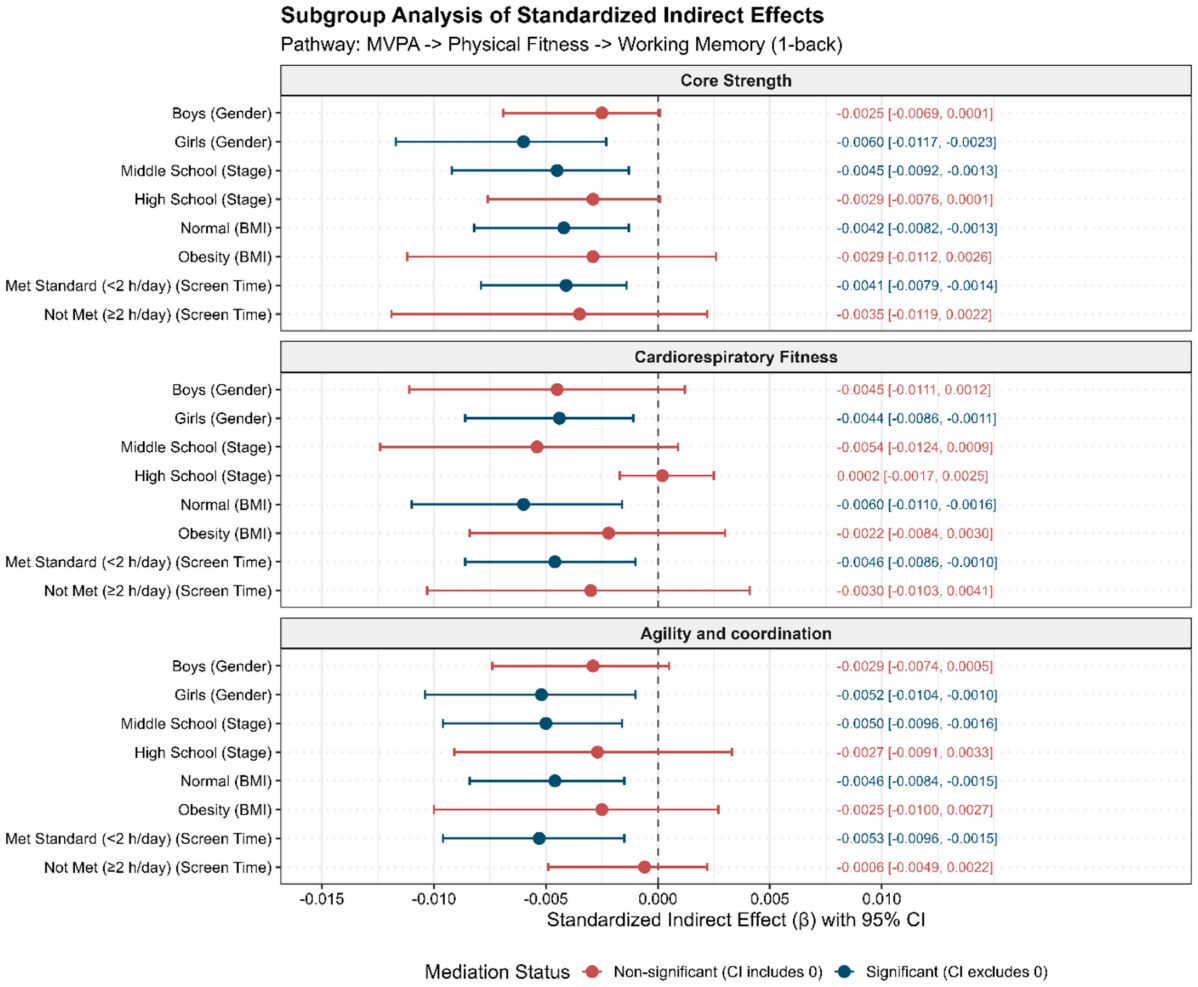
Forest plot of mediation effects across different subgroups.

Figure 5 illustrates the standardized indirect effects of MVPA on working memory mediated by PF, stratified by sex, educational stage, BMI, and screen time. Significant indirect effects through all three mediating variables were observed among female participants: core strength (β = −0.006, 95% CI: −0.012 to −0.002), cardiorespiratory fitness (β = −0.004, 95% CI: −0.009 to −0.001), and agility and coordination (β = −0.005, 95% CI: −0.010 to −0.001). No significant indirect paths were found among male participants (all 95% CIs included zero). Regarding educational stage, junior high students exhibited significant mediating effects through core strength and agility and coordination, while high school students showed no significant indirect effects across any PF components. Significant indirect effects through all three PF indicators were identified among adolescents with normal weight and those meeting screen time guidelines. However, these indirect paths were not significant among obese adolescents and those with excessive screen time, with all corresponding 95% CIs including zero.

## Discussion

The primary finding of this study was that multidimensional PF exhibited a significant suppression effect in the association between self-reported MVPA and working memory performance, as indexed by 1-back reaction time, among Chinese adolescents. Although these findings do not establish a causal mechanism, they are consistent with the possibility that PF may represent one pathway linking movement behaviors to cognitive outcomes, in line with the proposition that exercise-related neurocognitive benefits emerge alongside long-term physiological adaptations (34). By accounting for key lifestyle-related factors, including screen time, sedentary behavior, and sleep quality, the present analyses enabled a more nuanced examination of individual fitness components, extending the discussion beyond a generalized PA–cognition association toward the physiological processes that may underlie these relationships.

When compared quantitatively and qualitatively with findings from internationally recognized pediatric cohorts, our results identified cardiorespiratory fitness and agility–coordination as key mediating components, a pattern that closely aligns with evidence from the ActiveBrains project conducted by Esteban-Cornejo et al. (35). Their work demonstrated unique associations between these specific fitness attributes and greater gray matter volume in cortical regions critical for executive control, providing a plausible neurobiological basis for the mediation pathways observed in the present study. Converging evidence from the ActiveBrains and FITKids2 cohorts further supports this physiological pathway, indicating that distinct fitness components are independently and specifically associated with more favorable white matter volume (4). In contrast, our finding of a non-significant overall association between MVPA and working memory differed from results reported in the FITKids randomized controlled trial led by Hillman and colleagues, which demonstrated a clear causal pathway whereby participation in a supervised PA program enhanced executive control (36). This discrepancy may, in part, reflect methodological differences between the studies. Structured randomized controlled trials such as FITKids ensure intensive and objectively monitored participation in PA while simultaneously promoting cumulative physiological adaptations. By comparison, the present study employed a cross-sectional design and relied on self-reported PA measures. Although the instrument demonstrated acceptable test–retest reliability (*r* = 0.606), consistent with the characteristics of large-scale epidemiological investigations, the inherent recall bias and measurement variability associated with self-report assessments inevitably introduce statistical noise into estimates of actual behavioral exposure. From a sociocultural perspective, the sporadic MVPA typically accumulated within the context of intense academic demands and prolonged school-based sedentary routines among Chinese adolescents may be insufficient to yield immediate cognitive benefits. As proposed by theoretical frameworks that distinguish behavioral activity from physiological adaptation (37–39), movement behaviors may need to be performed with sufficient regularity and consistency to produce robust fitness adaptations, which may subsequently serve as a physiological pathway linking PA to EF.

The present mediation analyses identified agility–coordination and core strength as statistically significant mediators. Although these findings are consistent with previous research (40, 41), the significant mediating role of agility–coordination provides additional insight into the behavioral and physiological mechanisms underlying exercise-related cognitive enhancement. Unlike repetitive and relatively simple aerobic activities, agility–coordination-based movements are typically classified as open-skill activities (42), which are performed in dynamic and unpredictable environments and require continuous movement monitoring, inhibitory control, and strategic adjustment, thereby imposing substantial cognitive engagement (24). Although direct measurement of cognitive load is needed to empirically verify this interpretation, agility–coordination-related cognitive stimulation offers a plausible explanatory framework for the observed associations. The significant mediating role of core strength similarly warrants further mechanistic interpretation. From the perspectives of neurobiology and motor control, this relationship may be understood through the framework of cognitive–motor interference and dual-task paradigms. Core strength represents a fundamental prerequisite for postural stability; according to Schaefer’s ecological hypothesis of cognitive–motor dual-task performance, adolescents with lower core strength may need to allocate greater conscious attention and prefrontal cortical resources to maintain postural alignment and balance during daily activities (43). In contrast, higher core strength may facilitate more automatic postural control, thereby freeing limited prefrontal resources for higher-order EF s such as working memory. Furthermore, core strength training often involves complex movements requiring dynamic balance and multi-planar stabilization. Consistent with Diamond’s theory of motor–cognitive co-development, such coordinated motor demands may engage cerebellar–prefrontal circuits, providing a plausible neurophysiological pathway through which core stability may support cognitive performance (44). Collectively, these findings support the growing view that structured fitness-related components, rather than overall PA volume alone, may be more closely associated with executive control in adolescents.

In the heterogeneity analyses based on demographic and behavioral characteristics, a significant sex difference was observed, with the indirect pathway reaching statistical significance only among female adolescents. Two plausible explanations may account for this sex-specific pattern. First, it may reflect divergent trajectories of pubertal brain maturation, whereby females exhibit greater neurobiological sensitivity to training-induced adaptations in PF (45). Second, relatively higher baseline fitness levels among male adolescents may have introduced a ceiling effect, thereby attenuating the marginal cognitive benefits associated with further improvements in PF. This finding is consistent with neuroimaging evidence in adolescents indicating pronounced sex-specific associations between PA and brain structure (46, 47). Regarding educational stage, the mediation pathway was robust among junior high school students but not significant among senior high school students, a discrepancy that may be attributable to the more demanding psychosocial context of senior high school, where increased academic pressure and external stressors may offset potential cognitive benefits of PF. Future longitudinal studies incorporating relevant psychosocial covariates are needed to clarify these developmental dynamics. In analyses stratified by lifestyle behaviors and weight status, the indirect pathway was significant among adolescents with normal body weight and those meeting screen-time guidelines, but not among those with obesity or excessive screen exposure, suggesting potential boundary conditions of the fitness–cognition pathway in higher-risk subgroups. Although metabolic inflammation or prolonged sedentary behavior has been proposed as a mechanism that may attenuate cognitive benefits, such interpretations remain speculative and require further empirical validation in future targeted studies.

Despite these strengths, several limitations should be acknowledged. First, given the cross-sectional design, the mediation analyses should be interpreted as exploratory, and no causal inferences can be drawn. Second, although both objective assessments and self-reported questionnaires were incorporated, PA measures based on self-report remain subject to recall bias in the absence of accelerometer-based data. Additionally, estimating PA intensity relying on conventional MET values primarily derived from adult populations constitutes a methodological limitation, as adolescents generally exhibit higher resting energy expenditure and metabolic costs during locomotion. Third, PF was assessed at a single time point, precluding the capture of temporal changes. Fourth, pubertal status was not assessed in the present study, despite its relevance as a potential confounder in adolescents aged 13–18 years. Finally, the examination of multiple fitness components and cognitive outcomes may have introduced issues related to multiple comparisons. Future longitudinal and experimental studies are needed to validate and extend these findings.

In conclusion, the present study suggests that multidimensional PF plays an inconsistent mediating role in the association between PA and EF. This finding has important implications for public health policy. Future school-based physical education interventions should not focus solely on increasing the quantity of PA; rather, they should prioritize improvements in the quality of PF (i.e., specific fitness components) and the complexity of motor skill engagement, so as to more effectively promote cognitive development in adolescents.

## Conclusion

In this cross-sectional study of Chinese adolescents, PF components—specifically core strength, cardiorespiratory fitness, and agility and coordination—emerged as a statistically significant inconsistent (suppression) mediator in the association between MVPA and working memory. However, this indirect pathway showed clear subgroup heterogeneity, remaining significant only among female adolescents, junior high school students, adolescents with normal weight, and those meeting screen-time guidelines. These findings suggest that fitness-targeted strategies may complement volume-based PA promotion, although confirmation from longitudinal and intervention studies is still required.

## Data Availability

The raw data supporting the conclusions of this article will be made available by the authors, without undue reservation.
